# Do corporate governance structure and capital structure matter for the performance of the firms? An empirical testing with the contemplation of outliers

**DOI:** 10.1371/journal.pone.0229157

**Published:** 2020-02-27

**Authors:** Wang PeiZhi, Muhammad Ramzan

**Affiliations:** 1 Dean at School of International Trade and Economics, Shandong University of Finance and Economics, Jinan, China; 2 Ph.D. Scholar at School of International Trade and Economics, Shandong University of Finance and Economics, Jinan, China; The Bucharest University of Economic Studies, ROMANIA

## Abstract

A superlative combination of the Board of Directors (BOD) with diverse members is considered a sign of a good governance structure. Meanwhile, the key decision taken by BOD to make organizations profitable is the capital structure with the optimal mix of debt and equity. Unfortunately, previous literature has reported this relationship with a mixed trend, which may be due to research gaps in the statistical analysis. Moreover, it also shows that the relationship between them has not yet been fully predicted and can still be completely understood. This study contains time-variant and time-invariant variables, and these variables usually have an outlier’s problem. As we know that the OLS estimators are more sensitive to react adversely to this problem, yet we have not received enough evidence from similar researches that cares about it. Consistent with these arguments, this study focuses primarily on exploring the influence of corporate governance structure and the capital structure on firms' market-oriented and accounting-based performance, especially with the contemplation of outliers. Hypotheses have been evaluated using M-estimators and S-estimators of robust regression for 45 listed firms for the period from 2013 to 2017. The findings reveal that the governance structure of firms with BOD, independent director, institutional investors, audit committee and female directors accelerates its performance. Further, we find that the leverage ratio improves accounting performance, but it has a downward impact on the share prices of listed firms. Our study contributes to the prevailing literature by proving that the kind of governance structure that based on diverse expert members and a capital structure with a high volume of debt is of utmost importance to the performance of firms as a whole.

## Introduction

Corporate governance is a system that used to compose the best mix of the board members while governing and managing the firms properly and profitably. It is a vastly useful approach to manage and govern the organizations [1]. Notably, the board of directors (BOD) are to be considered the main players at a workplace for protecting the interest of all stakeholders [2]. Meanwhile, The BOD is a major stakeholder for making and implement the corporate strategic policies regarding all issues for smoothing the decision-making process [3]. In general, the nice strategic corporate governance practices give a way to know the vision of justifying the risk and optimizing performance with the aggressive and regulatory setting [4].

In particular, the effective combination of well-diversified and well-organized boards as a component of corporate governance depends primarily on two factors, the first one is its structure, and the second is its defined roles [5]. The board should assure the performance of all managers and employees with the regular check and provide a report to shareholders in the capacity of stewardship [6]. However, extensive literature exists about the defined role & the structure of the board, and its contribution to the performance of the corporations. The Board performs a wide range of business functions in two key areas, the overall performance of corporations and the overall compliance of accounting and other companies' standards [7]. The presence of independent directors on the board is better because they perform a central role in compliance with laws and protecting the shareowner's interest [8]. [9] Scholars said that relatively independent directors are more obedient to the rules and more concerned about the social responsibilities of corporations. The well-organized audit committees and audit standards of the corporations also contributed positively to compliance with the regulations [10]. Audit committees routinely direct departmental accounting performance in compliance with accounting reporting standards. The routine check on the accounting department helps to promote the financial performance of the firms. The scholars confirmed that the corporations should have the majority of independent directors, compensation, governance, and audit committees in a board for enhancing the financial performance [11]. However, BOD is also responsible for creating the best capital structure with the right combination of equity and debt as the firm's capital structure decides the future profits and growth of corporations [12]. Good governance and a balanced ownership structure help to create the proper capital structure, and thus corporations generate more profit. The high cost of debt financing puts downward pressure on corporations due to higher cash flows in the form of interest costs. Therefore, it is usually checked that relatively balanced or somewhat high equity financing is better for the growth and performance of organizations [13].

The structure of the board of directors is a group of people with different expertise who jointly direct the operations of the corporations [14]. The Literature supports members of a well-structured board of different skills, as well as an excellent mix of internal and external directors [15]. Similarly, studies have also proven that boards are comprised of independent and female board members, both of which have greater influence over the financial performance of large and small scale companies, but board members' age and board size did not contribute significantly to corporations' financial well-being [16]. A new investigation also claims that corporations are more diverse and the separation of chairman and CEO in a firm is more conducive to sustainable performance, while the presence of independent directors is detrimental to sustainable performance [17].

In the case of Pakistan, the scholars reported as the good structure of governance in the corporations helps to minimize the expected agency problem. Consequently, the agency cost also reduced and finally, it leads to an increase in the financial performance of the companies [18]. Similarly, the other studies also confirm a significant increase in the value of firm performance in the results of the good structure of governance [19]. However, the most recent studies recounted that firms in Pakistan try to build a diverse board and compose a good structure of governance at the workplace, but there is no certain evidence to establish the positive relation of good structure with the performance of the firms [20–21]. On the contrary, much of the evidence is overwhelming that poor board governance and corrupt formation have proven to be the root cause of business failures. The Mehrangate scandal is the biggest financial fraud in the banking sector in Pakistan's history. It is estimated at 5 billion frauds and is only due to corporate governance failure and corrupt practices of dishonest board members [22]. Another well-known case of Pakistan Telecommunication Limited (PTCL), is a state-owned corporation and offers telecommunication services for domestic people. The company suffered constant losses due to the corrupt administration and then the Govt. sell it at a loss of PRs. Twenty-three billion with reducing the per-share price from $ 1.96 to $ 1.65. Experts find that poor governance and the company's non-skilled board of directors are the only reason behind this public loss [23].

The only possible way for the administration to secure financial goals is through a structured board of directors that is protected from bad financial and capital management failure. Meanwhile, the fulfillment of the organization's financial goals depends on the best use of the resources available to the organization, so corporate governance is a really important consideration in this concern [24]. On the other hand, continuity, stability, and transparency of business through the use of good governance are equally important to maintain the economic growth of the nations. To ensure transparency, all stakeholders, including consumers, suppliers, and lenders, are also considered important in corporate governance. Certainly, transparency, accountability, justice, disclosure, and responsibility are the main goals of corporate governance [25].

All of this evidence claims that without good governance, there is a high chance of management fraud and failure, but it does not appear clear that a good structure for corporations' performance is truly guaranteed. This means that the relationship between corporate governance structure, capital structure and performance has not yet been predicted properly and can still be fully understood. Therefore, our research raises the question: Does corporate governance structure and capital structure have an impact on firms' performance? So, the ultimate purpose of this article is to investigate some evidence about the influence of the selected components of the board's composition (board of directors, independent directors, institutional investors, audit committees, executive directors and female directors) in corporate governance structure and capital structure on the share prices and financial performance. The present study plays an important role in the development of interdisciplinary discussions about which governance component, and the structure of the capital, plays a significant role in the improvement of share prices and firm performance.

The rest of the contents of the paper are as follows. The second part is about the theoretical foundation, reviewing the previous works of literature and the development of hypotheses, then the section of research design, methodology, and econometric tools followed by the result and discussion chapter. In the last, conclusion and references in the study are presented.

## Related studies review and theoretical foundation

The organization's timely decision-making procedures and rules guide the responsibilities and rights of various stakeholders in the shadow of the corporate governance structure. It is possible to create a good governance structure with the best BOD composition, and this is probably their first step. In any firm, the dominant decision-making body is the BOD and primarily protects the interests of the shareholders. Accordingly, the Agency and the Stewardship Theory have provided the board with professional content to fulfill this obligation. Another important duty of the board is to decide on the structure of the capital with the ideal level of a mix of both debt and equity, as this combination will determine the organization's future performance track. So the trade-of-theory of capital structure provides managers with guidelines in this regard. We are going to expound the relationship between corporate governance, capital structure and firm performance based on these three leading theories. Further, this section is intended to present the theoretical foundation of the study, review the relevant study literature and hypotheses development.

### The theoretical foundation of the study

The trade-off theory of capital structure suggested how the optimal level of debt and equity in the company's capital structure was chosen to derive maximum benefits from the tax shield as the interest expense is considered as tax-deductible expense [26]. In addition, it is also helpful in choosing the best financing level of debt and equity that will save the organization from financial troubles. Analysts say that blindly high leverage using excessive debt financing by the firms was the leading cause of the 1997 financial crisis [27]. An appropriate choice of capital structure with close care of the trade-off among interest tax shields and the cost of financial distress is the complete discretion of managers. Company owners (principals) hire managers (agents) to act in their best interests and take decisive actions that increase the wealth of shareholders. Agency theorists claim that the agents (managers) are liable for deciding and taking action for the firms’ financial betterment without regard for self-interest [28]. It is also argued that the financing decision using material and rational information for achieving the committed organizational goal by expressing professional behavior is also under the assigned duty of managers (agent) [29]. In reality, some agents may not purely act in the best interests’ of shareowners due to information miscommunication and opportunistic behavior, and this may lead to become a source of conflict among them. As a result, the agency relationship of agent and principal becomes an agency problem (conflict), and then the organizations face many losses called agency cost. The principle of separation of ownership and management in the business of the company results in the transfer of responsibility for all decisions on the shoulders of the management as they must have great information to achieve the goal of maximizing shareholders' wealth. The agency's theory helps to link the interest of managers and owners with the promise that there is no conflict of interest between the management and the firm owner [30]. The theory believes that management has no material relationship with the company, so they are always motivated by financial incentives to do what is best for the principal owners [31].

On the contrary, the stewardship theorists proposed that the relationship of agent and key owners is trustworthy so the managers assume to perform in the best interest to achieve the ultimate goal of the firms [32]. They argued that the management needs personal motivation for performing in the better interest of the organization by a willingness to achieve the maximum wealth for the owners [33]. The personal motivation more revitalizes through non-financial incentives like recognition, appreciation and authority promotion; however, agency theory believes in financial incentives. The theory also states that internal motivation/ incentives are better to drive management to adhere to rules and regulations as it is the best way for the firm's shareholders to reap maximum benefits [34].

Managers have more information than investors, and their actions, therefore, provide investors with an indication of the firm's prospects. Likewise, management assumes greater expertise in managing all financial and non-financial aspects of firms, including decisions about capital financing [35]. A large group of experts has suggested that corporate governance can be used to change the rules under which an agent operates and restores the interest of principals [36]. On the other hand, corporate governance also provides managers with a better understanding through which they utilize resources for maximum levels of production. Similarly, good governance in the workplace also provides guidance for optimal BOD composition for managing all firms' resources [37]. Therefore, many scholars claim that good governance helps to reduce the agency cost and promote the fiduciary and steward environment in an organization, so they gain more profit. Research on poor & good governance and firms’ profitability concluded as firms with relatively good governance have a strong profit margin due to stable leverage ratios [35]. Another study found that the application of principles of governance helps to increase the return from equity and return from assets of the company [38]. A recent study in Pakistan's context reveals that good governance structure helps to reduce agency costs and also controls its negative impact on firms' performance [21].

On the other hand, many studies have proven that poor or bad governance has a negative impact on firms' overall performance. It is also a failure of the management of the firms due to poor governance of the firms as the workplace governance assurance is the responsibility of the management [39]. The malpractice at the workplace has undermined the governance structures, supervisory control, and internal regulatory system of the organizations; it leads to raise the conflict of interest and agency cost that ultimately cause to lower firms' value [40]. Similarly, the Lehman Brothers case has been described as a failure of corporate governance due to its high leverage ratio. The leverage of Lehman Brother estimated the annual 30 times of its equity because they believed in high leverage high return philosophy, but CEO claimed that this failure just due to agency problems as company employees only Owned a small portion of the stock [41].

Meanwhile, some scholars stated that there is no magic to turning a bad manager into a good manager, but debt can play an important role [42]. Debt in leverage ratio may restrict the bad managers from vesting the owner’s equity and encourage managers to work more effectively [43]. Bad governance occurs due to the misconduct of managers to fulfill their duties and consequently fails to achieve sustainable development. As a result, it promotes agency problems and also reduces the capabilities of organizations. In the case of Pakistan, the study revealed that the lack of understanding, inadequately trained personnel (manager), deteriorating performance, mismanagement, and policies are the main causes of poor governance [44]. Pakistan Airlines (PIA) is the leading national airways corporation but has suffered financial losses over the past decades. It has been proven that the root cause of PIA's poor performance is its poor governance and corrupt administration [44]. In the recent trade era, Pakistani organizations also try to gain a foothold in global markets, but they have mostly failed due to poor governance [45].

The current literature examines various aspects of corporate governance, capital structure, and firm performance. For the ease of calculation and exposition, we divide the corporate governance structure into Size of the board of directors (BODs), independent directors, institutional investors, audit committees, female directors and executive directors. The leverage ratio is considered for the capital structure, and firm performance is determined by the ratio of return on assets (ROA) and share prices of the companies. In order to illustrate the problem under discussion and development of the hypotheses, we review all those studies in which corporate governance and capital structure assume an exogenous and studies where it is endogenously determined.

### Corporate governance structure and firms performances

The basic concept of corporate governance is to tell society that large organizations are well managed so that shareholders and lenders can feel free to invest in the organization [46]. It is also true that stakeholders' rights can be protected against any kind of mismanagement and corruption in local societies using the best practices of corporate governance. It is also used as a mechanism for running corporations when ownership and management are separated [22]. Similarly, it considers as vital for developing countries as these nations are able to achieve a high level of economic growth, stimulate savings, and build a credible economy through better management of industries. Besides, it is also equally important in an organization to raise the rate of investment and protecting the rights of minority stakeholders [47].

Numerous studies have evaluated the influence of corporate governance on stock returns as it is considered to be the key to moving the organization into a more comfortable and profitable environment. Setting up good corporate governance policies offers many benefits at various levels as it helps corporations avoid corruption, building and shaping a high level of shareholder value by shrinking financial risk [48]. It is, therefore, important that the corporation develops, executes, and maintains a stable governance policy. Empirical testing established a direct link to good governance in organizations with the firm's share prices, and they also determined that different levels of corporate governance had a different link with share prices and performance of the firm [49]. As a result, a study established that the application stage of principles and rules of governance to an organization leads to an increase in both equity and asset return [38]. In another study, the link of corporate governance score was measured with share prices of KSE-30 index listed companies of Karachi stock exchange. They found the direct positive link of the score of corporate governance with the listed companies' share prices [50]. An interesting study was conducted to measure the response of share prices to corporate governance score announcement results between 2007 and 2013, and they found a sharp positive change in share prices during the first announcement day [51]. Recent studies have focused on various aspects of corporate governance and its relationship with the sustainable development of the entire industry and economy. A study on governance and sustainable performance of firms have established that different component of governance in an organization cause sustainable performance [21]. Likewise, a study about corporations' governance and climate change management found that different governance mechanisms help effectively mitigate and implement climate change management strategies [52].

In this study, we consider following different characteristics of the board of directors suggested by agency theory and various mechanisms of corporate governance and its effect on firm performance for the development of the hypotheses of the study.

### Size of the board of directors

There are two schools of thought about the size of the board's relationship with the firms' performance. The first group of scholars argued that the larger board size is more efficient for companies to perform better because they have a larger amount of information for decision making [53–54]. While other groups emphasize the smaller size of the board, they have established that the smaller size of the board is more effective in decision-making, so it increases efficiency and leads firms to a higher level [55–57]. Many studies have taken the size of the board as exogenous and have established a positive influence on the return on assets, share prices and overall performance of the firms using OLS regression, fixed and random effect model, and DAE analysis for efficiency [58–59]. A recent study on the size of the board using the panel regression model found that the size of the board increases the financial performance of listed companies in emerging South Asian markets [60]. Accordingly, our first hypothesis is

**H1:** There is a positive relationship between the size of the board of directors with share prices and firms' performance.

### Independent directors

Independent directors are those who are not employees and have no material relationship with the firms. The extensive literature suggests that the presence of independent or non-executive directors helps to increase the value of firms with their independent view of policies [61]. It is also true that non-affiliated or external directors are vital to companies' sustainable financial betterment and development [62]. Meanwhile, the principles and code of the corporate governance emphasize on the balanced composition of board members and consist of independent directors [63]. A study acknowledged the board's independence as both exogenous and endogenous and found no positive association among outside directors and the economic performance of the firms. However, they found the positive influence of the size of the board on board independence [64]. On the other hand, the scholars also found the negative impact of external directors on performance because they found that the balanced size of the board's independence could lead to an increase in the value of these companies [60]. Though, the large number of independent directors does not guarantee the betterment of the shareholders' value. Scholars also found a positive and significant moderate relationship of the board's independence among CEO duality and performance using the dynamic GMM model [65]. In the case of developing countries, recent studies have used Panel OLS and GMM models and revealed that the board's independence proved better for firms' equity performance and economic performance [66]. Thus, our second hypothesis is

**H2:** There is a positive relationship between independent directors with share prices and firms' performance.

### Institutional investors

Institutional investors are those who pooled or collected money from individual investors to buy securities and other investments. They only have an investment relationship and influence in making firm decisions [67]. Experts argue that a large group of shareholders has more influence and control over the managers' activities, which in turn increases the value of the firms [68]. Over the past decade, institutional investors have become vital to improved performance with active monitors, and they have also been instrumental in increasing the value of shareholders by attracting more investments [69]. According to these arguments, several studies have found a positive and significant influence of active institutional shareholders on equity performance. They found that the apparently active behavior of institutional investors is more efficient, and relationships with firms' performance are bilateral [70]. Similarly, studies using fixed effect, random effect, and GMM model found that the institutional investors also have a positive influence when they are the second-largest investor in family-owned companies, and they also contribute positively to the financial performance of the companies in the early years [71–72]. One study claims that institutional investors are the leading cause of the financialization process for UK firms [73]. Recent studies on listed companies of the Pakistan Stock Exchange show a mix (positive, no) relationship between institutional investors and firms using the GMM model [74–75]. Meanwhile, our third hypothesis is

**H3:** There is a positive relationship between institutional investors with share prices and firms' performance.

### Number of audit committees

Audit committees are committees in organizations that are responsible for overseeing the performance of external and internal auditors with a regular scrutiny of the financial reporting process. Corporate governance considers audit committees as a mechanism and plays an important role in increasing public and regulatory interest [76]. It also plays a role in protecting shareholders' equity and interest and reducing the chances of fraudulent financial reporting. At the same time, the assurance of the integrity of the financial reporting system, internal control system, internal audit, and risk management should also be monitored by audit committees [77]. Further, the study revealed the positive role of audit committees in the performance of equity and earning per share, thus increasing the wealth of the firm's principal owners [78]. On the other hand, more detailed studies suggest that if an external director heads a committee, it leads to greater better performance than a committee headed by internal directors [79]. Likewise, the scholars claim that the knowledge, size, and independence of audit committees also help to increase financial performance [80]. According to a study, the structure of the Audit Committee meetings, the members, the frequency is positive while the size, the independent members of the Audit Committees have negatively contributed to the financial performance of the Pakistani firms [81]. From this perspective, our fourth hypothesis is

**H4:** There is a positive relationship between audit committee size with share prices and firms performance.

### Female directorship

Gender diversity at BOD is considered a positive sign because it helps to increase efficiency and independence. Therefore, it has been found that female directors, with their positive and proactive attitude, make a positive difference in the performance of firms [82]. Another study reported that female directors bring positive financial performance while they are not good for the firm's market-driven performance [83]. The presence of women in the boardroom is also important for the implementation of policies, rules, and regulations and for that reason, they make significant positive changes in the earnings of corporations [84]. Meanwhile, empirical evidence using GMM and multiple regression suggests that during crises, firms need more monitoring and unique advice, thereby the female representation in board help to improve the firm’s financial performance with new ideas [85]. At the board level, corporations have now tried to change the rules in different ways because many countries, including Norway, Spain, require listed companies to have at least 40% of BOD female directors [86]. A recent study of Pakistan-listed companies reveals that the presence of female members on the board is good for enhancing financial performance, but the number of female members is not necessarily related to performance [20]. Given that, our fifth hypothesis is

**H5:** There is a positive relationship between female directors with share prices and firms' performance.

### Executive directors

Indeed, organizations are independent of their owners with separate artificial existence but are not like other physical people. So it must act through the real physical people, and these people are directors. A group of directors called a board of directors, and they are not sometimes actually run all business activities by itself. Therefore, they give some of their powers and permission to others to act on their behalf and make an everyday decision; these people called executive directors [87]. They are also tasked with protecting corporate owners' assets and interests as a proxy for corporate governance [88]. They are also known as inside directors and have more real information than external directors. Likewise, if executive directors play an effective role in monitoring through appropriate information, the corporate governance structure is improved, which will increase the performance of firms [89]. In reality, the CEO appoints executives, and they are in his power and authority. So, because of their impending relationship With a CEO, internal directors cannot participate in the effective monitoring of the CEO. Therefore, a large number of executive directors do not guarantee better financial performance of the firms [90]. However, to the best of our knowledge, the evidence regarding the role of executive directors in the market and accounting performance is scarce and cannot be found in Pakistan's context. So, our sixth hypothesis is

**H6:** There is a negative relationship between executive directors with share prices and firms' performance.

### Capital structure and firm performance

Capital structure means the combination of debt and equity in the firm's total capital. This is considered as an important decision of financial managers in maintaining the competitiveness of the firms. In general, it helps to select the optimal level of debt and equity for the capital structure as the optimal level is key to minimize the cost of capital and maximize shareholder value with the best management of risk and return of the firms [91]. Likewise, using debt financing is meant to reduce the cost of capital as interest expense is tax-deductible so that debt financing can increase the value of the firm. So, different compensation schemes should be introduced in order to attract managers towards the organizational goal in a good corporate governance environment, as it is the responsibility of managers to make maximum capital structure [68, 92]. Scholars reported that the firms with 60% debt financing considered high leveraged firms and the tax shield had a positive contribution to their firms' earnings [93]. In contrast, many studies also found negative effects of leverage on firms' assets and equity earnings due to high financial costs [94]. A recent study suggests that high leverage is harmful to the accounting performance of the Pakistani listed firms, while product market competition is the best alternative to debt financing to align the interest of managers and owners [95]. Therefore, our seventh hypothesis is

**H7:** There is a negative relationship between financial leverage with share prices and firms performance.

### The rationale of the study

In this section, we summarize all the previous studies. They were focusing on two important issues of corporate governance—first, how a company's leadership structure can influence corporate governance and second, how the formation of a board of directors can impact sustainable performance. They used OLS, fixed effect, random effect, and GMM models as an econometric tool, and the results of these studies are contrary. After reviewing all prior literature, we can claim that no one explored the effect of the structure of corporate governance and the structure of capital on the financial and market performance of the firms with proper care of outliers. The study of governance consists of factors such as numbers, values, and percentage of observations. Meanwhile, if you contain such kinds of factors in your study, the problem of outliers, endogeneity, heteroscedasticity, and multicollinearity are common due to different units of observations. So our study is going to measure the ultimate objective of the study mainly with proper contemplation of outliers and all other discussed diagnostic problems simultaneously.

## Research design and econometric models

The research design is deductive, quantitative in nature and researchers are being positivist during this study. For our study, we used the firms' level data of listed companies of the KSE-100 Index of Pakistan Stock Exchange for the period 2013–2017.

### Sample, variables, and data

The study sample comprises of 45 listed companies were randomly selected from the KSE 100 Index of the Pakistan Stock Exchange, similar to the recent work of Aslam [96]. The corporate governance and capital structure used as predictor variables. Meanwhile, there are six components, including Board size, independent directors, institutional investors, audit committees, female directors, and executive directors were taken as the mechanism of corporate governance structure, and leverage ratio has been used as a proxy for capital structure. All of these measures of our predictors were inspired and adapted from the framework of Detthamronga et al. [97] and Khan & Subhan [20]. According to the previous literature, there are several proxy measures for firm performance, but in order to avoid the proxy specific biasness, we used share prices as a proxy for market-based performance and ROA ratio for accounting performance. Several studies on corporate governance have used these proxy measures for firm performance [92, 95]. However, we need to control other variables that are likely to affect the performance of the listed companies. The natural log of the total assets and the total capital of the firm were chosen as the control variables as they could affect the performance of the firm, which was suggested by Paniagua et al. [98]. The panel dataset has been collected with a total of 224 observations for the period 2013–2017, and this approach is in line with the recent work of Salloum et al. [99]. The rationale behind choosing this period is that no company has been closed, merged or acquired during this period. Another reason is that a 5-year timeframe is sufficient for consistent and robust results of this type of research [52]. The annual reports are utilized, which are downloaded from official websites of the companies and the official website of the state bank of Pakistan for the selected period. Additionally, Annual average share prices of listed firms were compiled from the official website of Yahoo Finance for the selected timeframe. The names, labels, and descriptions of all the included variables are listed in Table 1.

**Table 1:** Variables and description.

S#	Variable	Labels	Data Description
1	**Firm's Performance:** Share Prices	**FP** SP	The average annual price of a single share of the company
2	Return on asset	ROA	An indicator of how profitable a company is to its total assets.
3	**Corporate Governance:** Board of director	**CG** BOD	A group of individuals elected to represent shareholders.
4	Independent director	Ind_D	Who does not have a material relationship with the company and hired from outside.
5	Institutional investor	Ins_I	Which pools the money of individuals to purchase securities, real property or originate loans.
6	Audit committees	Ad_C	Overseeing the financial reporting and disclosure and nominated from the board.
7	Female directors	Fem_D	A female representative inboard of a company.
8	Executive Directors	Ex_D	Nominated inside members to act on behalf of other directors
9	**Capital Structure:** Financial leverage	**CS** Fin_L	This ratio represents the use of debt financing to acquire an additional asset.
10	**Control Variables:** Log Assets	**SIZE** Ln_A	Natural log of total assets of the firms.
11	Log Capital	Ln_C	Natural log of the total capital of the firms.

### Econometric models and methodology

Indeed, the panel data increases the degree of freedom and efficiency of the econometric models and also helps to control the unobserved heterogeneity between the cross-sections [100]. Similarly, fixed and random effects are more appropriate for panel data analysis because it calculates changes within the individual and the group. It also helps to eliminate the unobserved heterogeneity, and omitted variables bias problems in the data set, which is recommended by Berger & Patti [101]. First, we examine the different assumptions of OLS regression to achieve the ultimate objective of the study. For normality, the dataset contains more than 30 observations that are considered to be normal, which is suggested by Gujarati [102]. We check multicollinearity by a variance inflation factor (VIF), the autocorrelation is measured through the Durbin Watson test, and heteroscedasticity of the variance of the residuals tested through Breusch Pagan and Cook-Weisberg test. Another assumption of the OLS regression model is that residual term (e) and explanatory y (x) variables do not share linear relationships with each other. If this happens, then we said there is a problem of endogeneity [103]. In addition, we applied the OLS regression and created the residual term using the error-generating command on the software, and then tested the endogeneity between the predictors and residual term using the correlation analysis. Finally, we begin our experimental section using the following OLS regression equations:

SPit=α0+β1(CGit)+β2(CSit)+γZit+εit (1)

ROAit=α0+β1(CGit)+β2(CSit)+γZit+εit (2)

Here, SP stands for average annual share prices of all included firm i at time t, ROA shows the return on assets ratio, CG refers to corporate governance that we use for all selected components of corporate governance, and CS identifies the capital structure, and we use the leverage ratio for CS. Similarly, αo is the unknown intercept for each entity, β is the coefficient of explanatory variables, Z is a vector of firm-level control variables, and ε is for a residual term.

We then used the fixed effect (FE) estimator of the panel OLS analysis on our strongly balanced panel to control for the within-effect of the firms because it could bias the predictor or outcome variable. So, FE eliminates the time-invariant features within-group, and we can measure the exact effect of the explanatory on dependent variables [104]. If it does not, then there is the issue of endogeneity arises.

Subsequently, we used the GLS random effect (REGLA) estimator of the panel OLS model because it helps to protect our results from heteroscedasticity and autocorrelation problems as our model consist of time-variant and time-invariant variables, So this type of variable usually has heteroscedasticity and autocorrelation problem [21]. The rationale behind the random-effects model is that it is assumed that the variation across entities is to be random and uncorrelated with the explanatory or independent variables included in the model [104]. However, the following equations were used to measure the FE and GLS random effect estimators:

SPit=α0+β1(CGit)+β2(CSit)+γZit+ηi+νt+μit+εi (3)

ROAit=α0+β1(CGit)+β2(CSit)+γZit+ηi+νt+μi+εi (4)

Here, ηi means the firm fixed effect, which is incorporated in the proposed model specification to control for unobservable firm-specific and time-invariant heterogeneity; νt is for the time-fixed effect, which is used in the model specification to control for unobserved time-variant effects to all firms in the sample, μi is for between entity errors, and εi is for within entity errors.

Finally, we address the problem of outliers using leverage versus normal residual plots and examining the model's robustness. It is also true that outliers are also common when your model contains time-variant and time-invariant variables. All the estimators of the OLS model that we have already estimated are more sensitive to responding to outliers. So, there is a need to further examine the behavior of outliers and to determine the actual effect of the predictor on the outcome variables. Therefore, we have applied further robust regression, as this model is best to treat outlier [105].

Robust regression can be used in any case in which we use OLS regression [106]. After applying the OLS regression, we may find some outliers or high leverage data points. These data points do not indicate the required data entry or any other error. So we have absolutely no valid reason to ignore them from our analysis, then a robust regression is the best choice as it treats outliers like other normal data points with giving lower weight instead of ignoring them [107]. The following two estimators of robust regression are applied to treat the problem of outliers and test the robustness of the model. As we know, the OLS estimator is considered appropriate because it minimizes the sum of squared residuals.

$$\sum_{i=1}^{n}\;(Yi-Xi\beta i)2+\varepsilon i,\;\frac{\varepsilon i}{\varepsilon i}=0$$ (5)

The above OLS equation shows how the firm i observation of *Y* responds and covariates with *X*. β is an unknown vector and E(εi) is assumed to zero. Likewise, we replace the criterion of OLS estimator with a robust regression M-estimator which is proposed by Huber [108].

$$\hat{\beta }=\arg\;min\beta \;p\sum_{i=1}^{n}\;(\hat{\frac{Yi-Xi\beta i}{\sigma }})$$ (6)

Here, arg min is an argument used for assuming outcomes are as minimum as possible, p (.) is used for the robust loss function, (σ)^ is for error scale estimator, and this robust loss function minimizes the weighted sum of residuals. Further, the robust regression S-estimator has applied to examine which approach is best for dealing with our outliers and to give us a better idea of the robustness of the model. The following equation has been used for the measurement suggested by Rousseeuw & Yohai [109].

$$\hat{\beta }=\arg\;min\beta \;\hat{\sigma }\;(ri\;(\beta )\ldots \ldots \ldots \ldots rn\;(\beta ))$$ (7)

Here, ri (β) is equal to the *Y*i– *X*iβ and $\hat{\sigma }$ (r_1_ (β) ………… r_n_ (β)) is the scale of s-estimator which minimize the measure of dispersion 𝜎 of the residuals that is less sensitive to outliers than the variance.

## Empirical analysis and discussion

Descriptive statistics and correlation analysis of all variables of our study show in Table 2. The mean value of share price shows that the average per-share price of each involved firm is 149.40, and the average ROA value of 17.28 shows that this is the average return from assets of each incorporated firm. This means that every company listed on the Pakistan Stock Exchange generates an average profit margin of 17% of its total assets, which is a good indicator for the market. If we talk about the financial leverage ratio, it shows a value of 1.64, which means that the average financial leverage ratio of each firm is more than 1%. This shows that firms have taken on more risk, so this is a concern for the entire market as experts say the major cause of the financial crisis was excessive use of debt financing in 2008. As already mentioned, our study contained numbers and values that showed the least value of most of our variables to be zero.

**Table 2:** Descriptive statistics and correlation analysis.

Variables	Obs.	Mean	Max	Min	Std Dev.	SP	ROA	BOD	Fem_D	Ind_D	Fin_L	Ins_I	Ad_C	Ex_D	Ln_A	Ln_C
SP	224	149.40	1813.13	0.42	288.13	1.00										
ROA	224	17.28	560.70	-7.59	78.60	0.41**	1.00									
BOD	224	8.13	14.00	4.00	1.90	0.32**	0.24***	1.00								
Fem_D	224	0.25	4.00	0.00	0.62	-0.16*	-0.09	-0.09*	1.00							
Ind_D	224	1.67	6.00	0.00	1.27	-0.33*	-0.15*	0.15**	0.07	1.00						
Fin_L	224	1.64	45.32	0.01	6.07	-0.07*	-0.05**	-0.10*	-0.08*	0.04***	1.00					
Ins_I	224	4.84	18.00	0.00	4.23	0.48***	0.11*	0.20***	-0.09	-0.46**	-0.15*	1.00				
Ad_C	224	3.82	14.00	1.00	1.22	0.41***	0.16**	0.46**	0.04**	-0.10**	0.01**	0.22**	1.00			
Ex-D	224	1.44	4.00	0.00	0.88	-0.58	-0.23*	-0.33*	0.28**	0.41	0.08***	-0.58*	-0.28*	1.00		
Ln_A	224	31.34	70.18	2.27	46.89	0.12***	-0.01*	0.07*	-0.03**	-0.09**	-0.01**	0.05**	0.01***	-0.11*	1.00	
Ln_C	224	8.54	20.47	1.87	4.49	0.29**	-0.09**	0.16***	0.09***	-0.37	-0.14**	0.70***	0.08**	-0.55	0.101**	1.00

**Source:** The Author’s Investigation **Note:** Table 2 shows the results of the outcome of descriptive statistics and Pearson correlation analysis again the all included predictors and outcome variables at a significant level of **5%, *10% and ***1% respectively

Whenever we want to know the strength of the relationship between two or more variables, correlation analysis is often used. As presented in Table 2 below, there are three components of corporate governance, which include BOD, institutional investors and audit committees have a positive relationship with the performance of companies in Pakistan. The image of this positive relationship is presented that when these components of corporate governance increase, the share prices and returns on the assets of companies also increases. The other two characteristics of the board, such as independent directors and female directors, are negatively related to firms' performance. Similarly, debt financing is negatively related to the performance of firms because high leverage firms are at greater risk and benefit from it, which has a negative impact on share prices and profitability of the firm's assets.

Interestingly, the size of the board of directors is more strength of relation with share prices of the companies then ROA, the same is the case with the size of the audit committees and institutional investors of firms. Meanwhile, the final picture of the results indicates that the large size of the board of directors, the presence of institutional investors in the investment structure and the existence of audit committees in an organization attract investors to invest in and buy shares of companies. Therefore, all these components are more vital to the strengthened market-based performance of the companies.

### Impact of corporate governance structure and capital structure on firm’s performances

We begin with our main experimental section using the OLS, fixed-effect, and GLS random-effects models, as many scholars have suggested that these models are suitable for measuring the core objectives of the study [110]. The panel OLS is a suitable model because it minimizes squared residuals and improves the model's accuracy. Likewise, the fixed effect is an appropriate model where we want to constant the within-group properties of the firm. Whereas, GLS is more appropriate to encourage the likeness of the outcomes where we have time-variant and time-invariant variables [21]. Table 3 shows the results of our estimation and the Hausman test significance show that the fixed effect is more appropriate in terms of share prices, and the random effect is more appropriate in the case of ROA.

**Table 3:** The panel estimations: OLS, fixed effect, and GLS random effect.

	OLS Regression		Fixed Effect Model		Random Effect Model	
Variables	Share Prices	ROA	Share Prices	ROA	Share Prices	ROA
BOD	11.12407	10.7869***	20.39397*	6.681964***	21.55642**	0.167981***
Fem_D	1.30457	10.86408	-10.16841*	-6.616382	-32.27420	0.137022**
Ind_D	-19.39412	-11.6812**	-3.990607	-1.501721	-18.15761	0.167981
Ins_I	***19.72756	3.07393*	21.85203*	-2.509519	26.23736***	0.167981*
Ad_C	49.70365***	-3.70823	-0.941566	1.462444	18.88043	0.137022
Ex_D	***130.7111-	***23.73492-	2.13081-	1.78904	***102.227-	11.03537-
Fin_L	-0.67488	-0.26184	2.381147*	-0.128443	0.239219	0.137022**
Ln_A	0.00003	-5.96E-06	-1.88E-05	-3.07E-06	-4.95E-06	0.167981
Ln_C	-12.98144**	-8.20255***	13.09872	0.452118	0.146884	0.137022
Constant	104.791	50.44222	-224.3397**	-29.86626	-188.1412*	-26.04020
R^2^	0.4565	0.1965	0.795448	0.847420	0.167981	0.847420
Hausman					22.917077 (0.0035)	7.372056 (0.4971)
DW stat	0.987306	0.518993	1.532218	1.093232	1.283346	1.452223

**Source:** The Authors’ Investigation **Note:** Values represent the estimated coefficient at the statistical significance level of *5%, **10% and ***1%, respectively. Values shown in parentheses () represent the significance level of Hausman test statistics.

Table 3 reports the results of the panel OLS estimation for the selected predictors in case of share prices and ROA, respectively. The results show that independent directors and financial leverage have an insignificant negative, while executive directors have significant negative effects on share prices. In contrast, BOD and female directors are positively associated with share prices, but the results are not significant. However, institutional investors and audit committees are positively associated with the 1% significance level. The value of R2 states that approximately 46% of the company's share price changes have been explained by corporate governance and capital structure selection. In the case of ROA, the results show that BOD and institutional investors have a positive relationship with ROA at 1% and 10% significance levels, respectively. Independent directors and executive directors are negatively and significantly associated with ROA, while financial leverage can also have a negative impact on ROA, but the results are not significant. Meanwhile, the R2 value represents only 20% of the ROA of firms defined by all predictors.

Then, Table 3 presents the results of the fixed effect and GLS random effect estimation of panel OLS. We only explain the results of estimations, which is reported as more appropriate by Hausman test significance value. The results of the fixed effect model show that the coefficient value of BOD is positive and statistically significant at the level of 5% in case of share price, it indicates a positive and significant influence of BOD on share prices of listed firms. Similarly, the findings also confirm a positive and significant relationship between institutional investors, financial leverage and company share prices, but the results of financial leverage are against our study hypothesis. Our results also similar to the arguments of Dalton & Dalton [111] and Garcia et al. [112], but the result is against the argument which is given by Mak & Kusnadi [113] that the negative relationship between BOD size and firm performance.

On the other hand, findings reveal that female director have a significant negative association with market-based performance. Likewise, independent directors and audit committees also have a positive but insignificant relationship with share prices. The R^2^ value indicates a variation of 80% in the share prices described by the corporate governance and capital structure components.

In the case of ROA, the GLS random effect model is more appropriate because the Housman test indicates it. The results indicate that BOD, female directors, institutional investors, and financial leverage has a positive association with ROA at 1%, 5%, 10%, and 5% levels. This result is similar to the findings of Detthamronga et al. [97]. However, executive directors have an insignificant negative association with the returns from assets of the listed firms. The results of independent directors and audit committees also share a positive but insignificant association with ROA. About 85% of the variation in the ROA is described by all the predictors included in this study.

Most of these results are discouraging because they are not in line with our developed hypotheses. This situation is ultimate of concern and points to something wrong with our variable observations. Subsequently, we performed various diagnostic tests of the panel OLS regression and found that our data contained outliers in observations. The following are the results of the OLS diagnosis.

The VIF test show in Table 4 is the test of multicollinearity assumption of the classical regression model. Multicollinearity is a problem that occurs when explanatory variables of the model share a linear relationship, and it may disturb the results of the regression model [20]. If the value of VIF is less than 10 or 1 / VIF is greater than 0.1, we can say that there is no strong correlation between all predictions. Thus, the results of our analysis reported that no predictor with linear relationships is found. After panel OLS regression, we tested heteroscedasticity of the variance of the residuals using Breusch Pagan, and Cook-Weisberg test and results indicate that the problem of heteroscedasticity exists in the model. However, further testing through the GLS random effect assist in resolving the problem of heteroscedasticity and autocorrelation, and the results are also somewhat improved.

**Table 4:** The variance inflation factor test of multicollinearity.

Variables	VIF	1/VIF
BOD	1.59	0.627149
Fem_D	1.29	0.776240
Ind_D	1.56	0.642380
Fin_L	1.04	0.961221
Ins_I	2.48	0.402455
Ad_C	1.38	0.725112
Ln_A	1.03	0.975016
Ln_C	2.21	0.451995
**Mean VIF**	1.65	

**Source:** The Authors

The next step is to test the exogenous assumption of the OLS regression, for which we apply the OLS model and generate the residual term through the automated software command, and then run the correlation test between the piece of great information and explanatory variables.

Table 5 shows the results of a test for the endogeneity problem. The OLS estimator is also sensitive if the error term of the model has a strong relationship with the predictors. The results of the correlation analysis show that not even a single predictor is related to the residual terms. Consequently, we move to our next diagnostic test, which is an outlier detection test in a model.

**Table 5:** The correlation analysis for testing the endogeneity problem.

Variables	Dependent variable: ROA	Dependent variable: Share Prices
	Residuals	Residuals
BOD	0.0000	0.0000
Fem_D	0.0000-	0.0000-
Ind_D	0.0000-	0.0000
Ins_I	0.0000	0.0000-
Ad_C	0.0000-	0.0000-
Ex_D	0.0000-	0.0000-
Fin_L	0.0000-	0.0000
Ln_A	0.0000-	0.0000
Ln_C	0.0000	0.0000

**Source:** The Author’s Investigation

**Fig 1:**
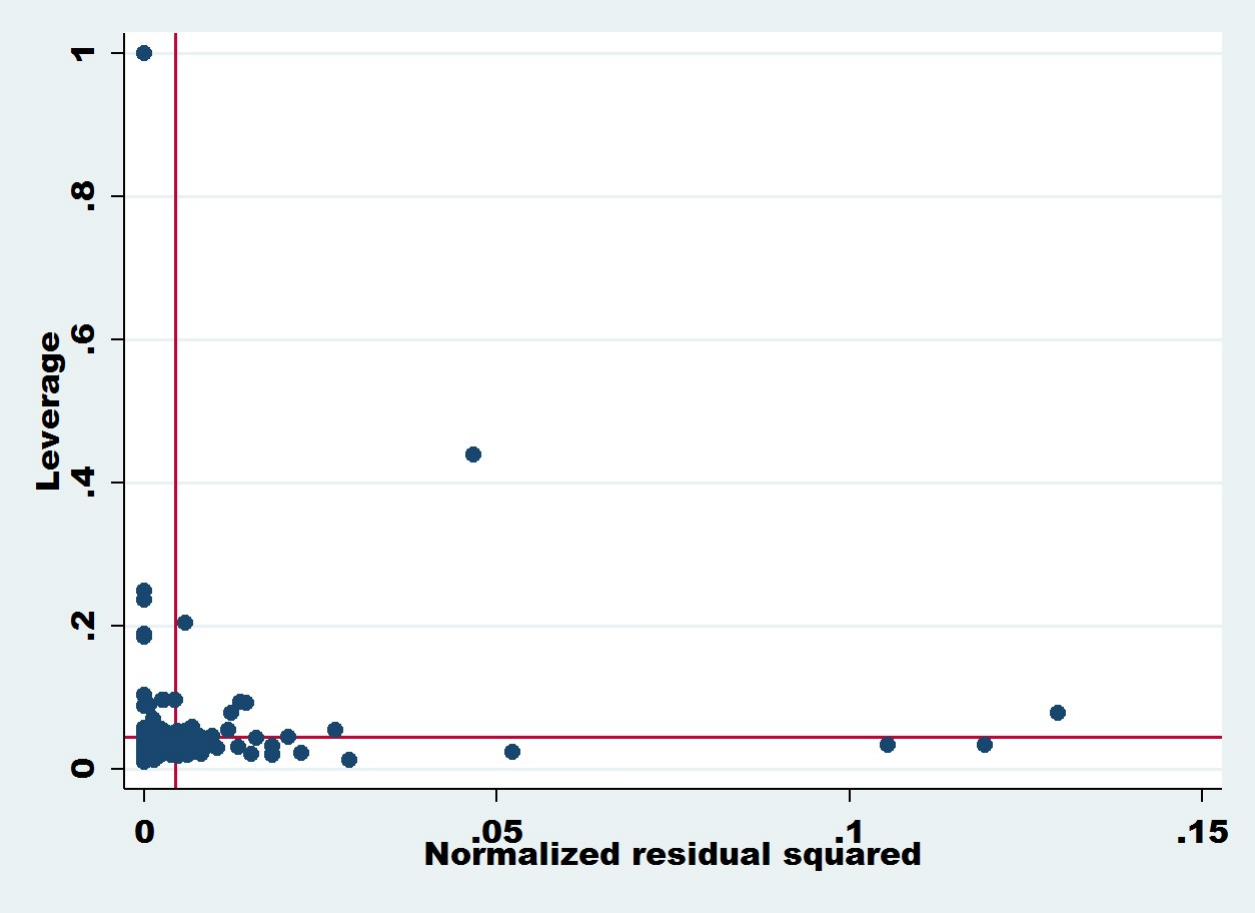
The diagnostic outliers plot using leverage versus normalized residual squared. **Source:** The Authors’ Investigations.

**Fig 2:**
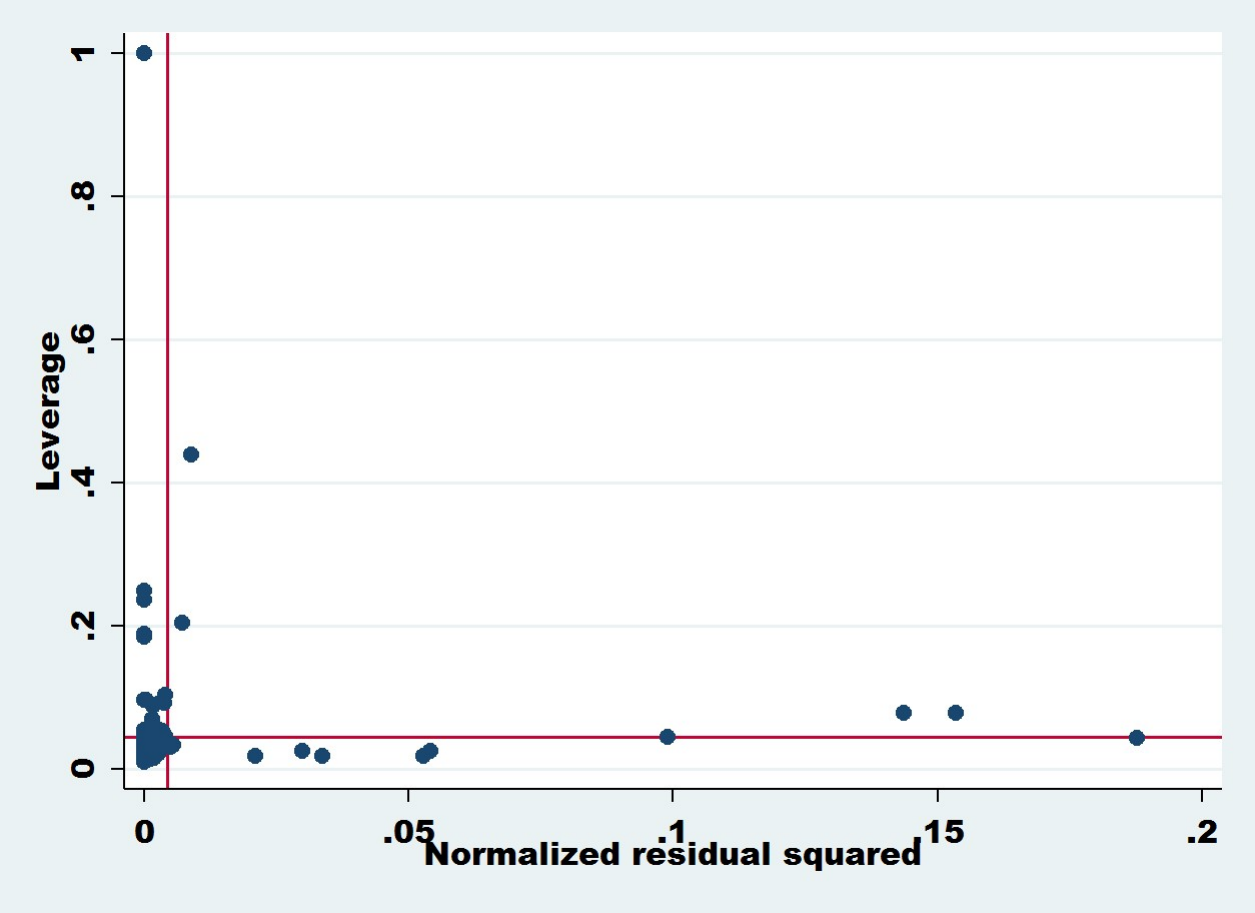
The diagnostic outliers plot using leverage versus normalized residual squared. **Source:** The Authors’ Investigations.

**Dependent Variable: Share Prices:** Figs 1 and 2 show the leverage versus normalized residual squares plot and indicates information about the outliers in our dataset. The outliers are observations that have high residual values, so this may be due to unusual values in the predictor variables. Leverage is a measure of how far an explanatory variable deviates from its mean value. Any observation of predictor variables with extreme value means it has a higher leverage point; a higher leverage point has more impact when we measure the regression coefficient [114]. Extreme values near the Y-axis are called vertical outliers and observations with extreme values near the X-axis are known as bad leverage. The bad leverage points can affect both slope and intercept measurement, while vertical outliers affect the intercept measure of the regression model [115]. In our case, both graphs (Figs 1 and 2) show that there is an outlier’s problem, which means we need to overcome this problem using an outlier-based model that is robust regression. A small number of outliers can adversely affect the results of the entire model as the OLS estimators are more sensitive to this problem. Therefore, the estimation of the OLS model is disturbed when extremes values are found in our model observations.

**Dependent Variable: ROA:** So the best way to avoid these extreme values is to apply robust regression to calculate the true value for slopes and intercept. Meanwhile, it is good to use this approach as it allocates less weight to outliers and consider to be an observation rather than being ignored [116]. Finally, we move on to our central analysis approach, and the results are listed in Table 6.

**Table 6:** The robust regression: M-estimator and S-estimator.

	M-Estimator-Robust Regression		S-Estimator-Robust Regression	
Variables	Share Prices	ROA	Share Prices	ROA
BOD	2. 27319**	0.02645***	0.00271**	0.02038***
Fem_D	6.58585***	0.03724*	1.24943*	0.024672*
Ind_D	1.17178*	0.00333*	0.86030**	0.00144*
Ins_I	0.49505**	0.00762**	0.37894*	0.00269*
Ad_C	0.73481	0.00505**	0.64495**	0.02155**
Ex-D	1.87576-	***0.05298-	-3.77892***	-0.01162**
Fin_L	-0.09677*	0.01506***	-0.13149*	0.01822***
Ln_A	3.38322***	0.02711***	8.99E-05***	0.09495***
Ln_C	-3.51381***	-0.03202***	-0.02850***	-0.08435***
Constant	-7.18288*	-0.02566	10.42898**	-0.23512***
RMSE	4.8871	3.6824	11.3304	7.1444
R-Squared	0.4942	0.8777	0.2282	0.3459

**Source:** The Authors’ Investigation **Note:** The RMSE stands for root mean square errors and all values in the table represent the estimated coefficient at the statistical significance level of *5%, **10% and ***1%, respectively.

We have applied two robust regression estimates to measure our principal objectives of the study and to test the validity and robustness of the models. Table 6 indicates the results of both M and S estimators of robust regression against both outcome variables. The results reported that the BOD has a positive association with the share prices of listed firms in Pakistan at a 5% significance level. Similarly, BOD also has positive effects on return on assets at a 1% significance level, but the coefficient values are better in the case of M estimator. This result is consistent with our first hypothesis and also endorse the findings of Aslam, Kalim, & Faza [21]. It has made it clear that listed firms should increase the size of the BOD, as it is the best way to increase market-based and accounting performance. The female members on board also have a positive and statistically significant influence on the share prices and ROA. However, the influence of female members on share prices is greater than ROA, which indicates that a board consisting of female members acts as an accelerator in the share prices of companies.

Further, it shows that if 1% of the female members on the board increase, this increases the company's share price by approximately 7%, which is also endorsed our hypothesis. Independent directors have a positive and significant relationship with both the maker-based and accounting-based performance of listed companies. The results of the M estimator are more robust, and the findings are similar to those of Bhagat [110]. However, the participation of independent directors depends on the authority and accountability that granted to the board by investors in all important matters. In general, independent directors play an effective role in enhancing shareholder wealth by protecting firms from various operational corruption in developing countries.

Institutional investors are considered a great patrol in the company's early years when companies issued initial public offering (IPOs). One study found that institutional investors contributed about 36% to first-year share prices and a 42% increase to second-year shares prices of the company [84]. Similarly, our findings also reveal that institutional investors have a positive and significant impact on the performance of listed companies in Pakistan. However, share prices are more likely to be affected by institutional investors as the average 1% increase in institutional investors increases the share price by 49.5% and 37.8%, respectively. The results also instructed companies to organize discipline independently for institutional investors who are more likely to enhance their company's market-based performance. The presence of experienced audit committee members will reduce financial mismanagement and increase quality supervision. As such, having experienced members of the audit committee should be a key priority for firms [117]. The results of our study reported that the size of audit committees has a positive association with the share prices and ROA of the companies. This means that a large number of committees are guaranteed to protect companies from financial mismanagement and therefore increase efficiency. Likewise, the results confirm the hypothesis of the study and indicate that these committees are also a positive symbol for companies' market appearance.

The results of executive directors indicate that they have a negative and statistically significant relationship with the overall performance of companies. The findings also endorsed our sixth hypothesis and the estimation of other scholars [90]. A possible explanation for this result is that the executive directors work under the influence of the CEO and because of this they are unable to perform with professional loyalty. In addition, if firms develop better strategies for the independence of executive directors, they will bring about more positive changes in firms' performance as they always have more information within them. The overall results of the corporate governance board composition and mechanism suggest that if a diverse board is created with independent and female directors, and there is a mechanism for governance of institutional investors and audit committees at the workplace, then those companies market-based and accounting performance should be improved.

The finding for a proxy of the capital structure reveals that financial leverage has a negative influence on companies’ share prices at a 10% significance level. It indicates in both estimation that the 1% increase in a financial leverage ratio of companies causes to drop the share prices at the rate of 9% and 13%, respectively. These results validate our hypothesis number seven and the findings of other scholars [118].

However, in the case of ROA, results depict that an increase in the debt financing causes to increase the profitability of the firm's assets. Therefore, we fail to accept our hypothesis of a negative association between financial leverage and ROA. The leverage ratio of firms reflects the different associations with both of our outcome variables. This could be due to two reasons. First, the high rate of debt financing in the capital structure reflects the risky image in the market, so investors do not rush to show interest in investing; as a result, its prices fall. Second, Interest expenses on debt are tax-deductible, so even more debt can increase the profitability of a company's assets.

The results of the robust regression M estimator are relatively more efficient because it shows lower values of the root mean square error (RMSE) and higher R2 values. As results reported, all of our predictors explained a 49% variation in share prices for listed companies and an 87.7% variation in ROA. Both estimates in Table 6 replicate almost similar results in terms of sign (+/-) of coefficient and significance that indicate the robustness of this model. Meanwhile, we accept almost all of our hypothetical ideas of study which indicate that we have achieved what we have decided to achieve, which is the validity of this model. The overall results of the study show the positive and significant impact of corporate governance structures on share prices and ROA. However, the capital structure has a positive affection on accounting performance and a negative association with market-oriented performance.

## Conclusions and practical implications

This study identifies a problem that is never properly addressed and treated by anyone while researching corporate governance. The focused problem is outliers, so the main objective of our study was to investigate the role of corporate governance structure and capital structure in the market-based and accounting-based performance of the listed companies with proper care of all assumptions and especially with regard to outliers. As already discussed, OLS estimators are more sensitive to outliers, and what we have studied in this research usually has an outliers value in their observations. It means that if we want to reveal the factual relationship among these variables, then this problem should be addressed; otherwise, we are unable to explore the true picture. For testing the hypotheses, the study obtained the latest data from 2013 to 2017 for the 45 listed firms randomly selected from the KSE-100 Index of the Pakistan Stock Exchange. To do this, first of all, we examined all assumptions, applied the panel OLS, fixed and random effect models, and found that there was an outlier’s problem.

The findings of M and S estimator of robust regression reported that the size of BOD, independent directors, female directors, institutional investors and audit committees have a positive and significant influence on the market and accounting performance of listed firms. Whereas, in the case of executive directors, we have been found that it has negatively related to performance. These results are in line with the claim of the agency's theory that the independence of directors, carefully selected board members (female board members) and monitoring through audit committees positively contributed to the firm performance. Certainly, these results also provide a guideline that corporations should work carefully while composing the board with diverse members and incorporate different resources to attract directors' interest toward the core purpose of the firm. In practice, inside (executive) directors have more in-depth information than outside (independent) directors, but they are mostly under the loyalty of CEOs, which is why they unable to perform their true role for the betterment of firms. Therefore, corporations should formulate strategies for the independence of executive directors, so surely they will lead to more positive changes in performance than independent directors. Overall, we found a positive and significant association of corporate governance structures, comprised of independent, female board members, institutional investors, and audit committees with share prices and ROAs of listed companies. Indeed, the capital structure is the main decision of the board, and the best mix of capital will be able to add positivity to stable firms’ performance.

We have found that the ratio of leverage is a negative and significant association with share prices, whereas this relationship is positive in terms of ROA. This suggests that high debt financing in the capital structure creates a negative image for investors in the market; investors refrain from buying the investment of these companies and consequently, the prices are dropped. On the other hand, we are unable to find evidence about the negative association between leverage ratio and ROA because they showed a positive and significant relationship. One possible interpretation of this association is that interest on loans before paying taxes is always excluded from the income of the companies, thus increasing the earnings of the corporation's assets. This means that managers should be aware of the special effects of debt financing when setting up a capital structure. Otherwise, it can have a profound impact on firms' share prices. The overall findings are in favor of our hypothesis of the study and endorsed the findings of agency theory and the trade-off theory of capital structure.

The practical implication of the study is that the industrial sector of any country plays an important role in the prosperity of the nation, and similarly, the governance in corporations play an important role in the prosperity of the firms, they are interconnected with such manners. The whole responsibility, in this case, lies on the shoulders of managers, they should decide technically about the best mix of debt and equity financing based on current and prospective market conditions and not on personal will. The study provides evidence that agency problems affect the decision of managers about capital structure, and it will directly affect the performance of the firms in terms of agency cost. Managers should reduce agency costs to improve firm performance, and this is only possible if they assure a good corporate governance structure in the workplace.

This article contributes to the literature by adopting a number of theories that understand the interactions examined in the study. However, the agency's theory is most widely used when studying corporate governance because it provides a rational basis for developing hypotheses. Logically, the findings of this article provide researchers with a new field of concept to study the relationship between corporate governance and firm performance in two main ways. First, the concept of the outlier and its role in disturbing the results of the study and its solution. Second, as we know, the strong relationship between explanatory variables and residuals shows the problem of endogeneity, but no one tries to detect it similarly. Therefore, our study provides a new way to detect the problem of endogeneity. However, there are some limitations that future researchers need to overcome. First, our study used only five years, with a total of 225 observations, with different time periods likely to yield different results. Second, we have conducted this study on the reporting standards used by companies listed on the KSE 100 Index of the Pakistan Stock Exchange and by the Pakistan industry and may differ from other countries.

## Supporting information

S1 File Data series. (XLSX)
